# Subjective cognitive decline in healthy older adults is associated with altered processing of negative versus positive feedback in a probabilistic learning task

**DOI:** 10.3389/fpsyg.2024.1404345

**Published:** 2024-07-10

**Authors:** Siri-Maria Kamp, Ricarda Endemann, Luisa Knopf, Nicola K. Ferdinand

**Affiliations:** ^1^Department of Psychology, Trier University, Trier, Germany; ^2^Department of Psychology, Bergische Universität, Wuppertal, Germany

**Keywords:** cognitive control, feedback processing, event-related potentials (ERPs), subjective cognitive decline (SCD), feedback-related negativity (FRN)

## Abstract

Older adults who worry about their own cognitive capabilities declining, but who do not show evidence of actual cognitive decline in neuropsychological tests, are at an increased risk of being diagnosed with dementia at a later time. Since neural markers may be more sensitive to early stages of cognitive decline, in the present study we examined whether event-related potential responses of feedback processing, elicited in a probabilistic learning task, differ between healthy older adults recruited from the community, who either did (subjective cognitive decline/SCD-group) or did not report (No-SCD group) worry about their own cognition declining beyond the normal age-related development. In the absence of group differences in learning from emotionally charged feedback in the probabilistic learning task, the amplitude of the feedback-related negativity (FRN) varied with feedback valence differently in the two groups: In the No-SCD group, the FRN was larger for positive than negative feedback, while in the SCD group, FRN amplitude did not differ between positive and negative feedback. The P3b was enhanced for negative feedback in both groups, and group differences in P3b amplitude were not significant. Altered sensitivity in neural processing of negative versus positive feedback may be a marker of SCD.

## Introduction

The term “subjective cognitive decline” (SCD) refers to a condition in which older adults subjectively perceive and worry about a decline in their cognitive function without fulfilling the criteria for a neurodegenerative or other neurological or psychiatric disorder as diagnosed by neurocognitive testing (Jessen et al., 2014). This condition has recently sparked interest, because older adults with SCD are at an increased risk for a dementia or mild cognitive impairment (MCI) diagnosis at a later time (Mendonça et al., 2016). Nevertheless, individuals with SCD compose a heterogeneous group, as the vast majority does not develop dementia (Mendonça et al., 2016). Hence, this may be a potential target group for early risk assessment and diagnosis, or even interventions aimed at the prevention or very early treatment of dementia (Smart et al., 2017). Due to its clinical relevance, it is important to better understand the etiology and correlates of SCD.

Neural activity may be a more sensitive measure of subtle neurocognitive changes than neuropsychological testing. Most of the – presently still limited – research on functional neural changes in SCD has used fMRI. The majority of studies comparing fMRI activity at rest (e.g., Dillen et al., 2017; Verfaillie et al., 2018) or during cognitively challenging tasks (e.g., Hayes et al., 2017) have indeed revealed differences in brain activation patterns in persons with vs. without SCD; however, the specific patters have been heterogeneous (Viviano and Damoiseaux, 2020; Parker et al., 2022). Some studies have suggested that individuals with cognitive complaints, despite unimpaired behavioral performance, show over-activations in several regions including prefrontal and anterior cingulate cortex (Dumas et al., 2013). Hence, compensatory over-activation of cognitive control systems due to inefficient neural signal processing may be a typical feature of SCD (Viviano and Damoiseaux, 2020). In the present study, we used the event-related potential (ERP) technique to capture potential changes in cognitive control processes in individuals with SCD at the neural level. ERPs are derived from the ongoing EEG signal and are a cost-effective tool for capturing task-related brain signals with a high temporal resolution. However, little prior research has examined ERPs indexing cognitive control processes in SCD, a gap that the present study aimed to fill.

### Cognitive control and performance monitoring in SCD

Altered cognitive control processes and performance monitoring may potentially be a marker of SCD. Accordingly, a mechanistic model of Mizuno et al. (2018) proposes that in early stages of (subtle) cognitive decline, a compensatory neural overactivation may lead to a discrepancy between the actual effort in completing a task and expectancies based on past experiences. This may lead to a prediction error signal, which is in turn resolved by adjusting one’s future expectations. In other words, individuals who develop a worry about their own cognitive function declining (i.e., SCD) may differ from individuals who do not, in the manner in which feedback from the environment or from internal feedback cycles is processed or evaluated.

Furthermore, individuals with SCD might show a processing bias toward negative performance feedback. That is, while most individuals may attribute small everyday mistakes (such as the temporary inability to retrieve a name or word finding difficulties) to a normal process that is not worrisome, some may pay stronger attention to such lapses and evaluate them as evidence for cognitive decline. In the latter case, the repeated experience of cognitive lapses and the greater significance attributed to these lapses may contribute to the subjective experience of a worrisome cognitive decline beyond the typical age-related development (i.e., SCD). An attentional bias toward negative feedback and/or a bias in the subjective evaluation of negative feedback, may hence be a feature of SCD.

In sum, hyperactivity in performance monitoring systems and/or a processing bias towards negative feedback may be a feature of SCD. Somewhat speaking against this idea, Buckley et al. (2016) reported that error awareness in a Go/NoGo-task was not directly associated with SCD symptoms. However, depressive symptoms mediated the relationship between the two variables: Higher error awareness was associated with stronger depressive symptoms, which in turn was associated with stronger SCD symptoms. This is in line with the fact that individuals with SCD typically score higher in depression and anxiety (Jessen et al., 2014). However, not all stages of performance monitoring and feedback processing necessarily depend on conscious awareness, and it may be beneficial to capture indicators of performance monitoring that are independent of awareness. To this end, in the present study, we examined feedback processing in individuals with vs. without SCD on the neural level by recording event-related potentials (ERPs).

### Feedback-related event-related potential components

The ERP components elicited by performance feedback include the feedback-related negativity (FRN) and the P3b (e.g., San Martín, 2012 for a review). These two ERP components were hence recorded as main variables of interest in the present study and are briefly introduced in the following.

The FRN is a negativity that peaks at fronto-central electrodes 200–350 ms after informative feedback about one’s own performance, or about a task outcome (Walsh and Anderson, 2012). Its amplitude varies with the extent to which an individual’s expectancies about an outcome are violated (Walsh and Anderson, 2012). The FRN is often larger for negative than positive feedback in simple behavioral tasks (San Martín, 2012), but can be enhanced for positive feedback when it is unexpected (Oliveira et al., 2007; Ferdinand et al., 2012), in part depending on the feedback’s goal relevance (Ferdinand and Kray, 2013; Severo et al., 2020). Therefore, the FRN may index a reinforcement learning signal, reflecting an outcome prediction error (e.g., Sambrook and Goslin, 2015).

The magnitude of the FRN may be sensitive to an individual’s attentional or motivational bias. Thus, a higher FRN amplitude to negative (relative to positive) feedback has been reported for individuals who show a higher extent of negative emotionality (Santesso et al., 2012), stronger depressive symptoms (Keren et al., 2018), or stronger anxiety (Tobias and Ito, 2021). However, prior results are not entirely consistent. For example, Gao et al. (2024) found no association between affective symptoms and FRN amplitude. Nevertheless, it appears that a processing bias toward negative information may go hand in hand with an enhanced FRN to negative feedback.

The P3b presumably reflects a context updating signal for task-relevant events (Donchin, 1981), and in feedback tasks follows the FRN with a typically centro-parietal maximum, thus reflecting a higher-order feedback processing stage (e.g., San Martín, 2012). Prior studies have reported a sensitivity of the P3b to feedback valence (e.g., Stewardson and Sambrook, 2020), but stronger determinants could be the magnitude or relevance of a reward (Meadows et al., 2016; Ferdinand and Czernochowski, 2018; Ferdinand, 2019). Notably, the P300 also correlates with affective symptoms, being typically reduced in individuals with depression (e.g., Klawohn et al., 2020).

### Feedback-related components of the event-related potential in older vs. young adults

Since SCD is a construct that is observed in older age, it is important to first address how aging affects the ERP variables of interest, the FRN and P3b. Healthy aging is associated with altered feedback processing and impaired learning from feedback (e.g., Ferdinand and Czernochowski, 2018; Cutler et al., 2022). Prior studies have reported reduced FRN amplitudes elicited by negative, relative to positive feedback in older adults (e.g., Eppinger et al., 2008; Pietschmann et al., 2011; Ferdinand, 2019; West and Huet, 2020), although some studies have shown similar valence sensitivity of the FRN across age groups (Ferdinand and Kray, 2013). While some have interpreted the former pattern as a processing bias, i.e., a stronger inclination to rely on positive rather than negative feedback in older adults (Eppinger et al., 2008), this pattern may sometimes be alternatively explained by the participants’ expectancies regarding their own performance, especially when the age groups differ in performance (Ferdinand, 2019).

Regarding the P3b, the typical finding is an amplitude reduction in older adults, which is accompanied by a “frontal shift,” presumably indexing a neural processing inefficiency or the compensatory recruitment of frontal areas (e.g., Kamp, 2020). Notably, patients with Alzheimer’s disease show a reduction in P300 amplitude beyond the typical age-related effect (Hedges et al., 2016). The P300 has thus been proposed as a marker of neuro-cognitive resources across the lifespan (van Dinteren et al., 2014).

In an exemplary study that is the basis for the present study, Ferdinand and Hilz (2020) asked young and healthy older adults to sort objects into one of two hypothetical moving trucks. Participants had to learn the correct assignment using probabilistic performance feedback. Both groups showed evidence of learning the correct assignment over time, although the older adults learned at a slower rate. Importantly, the FRN was differentially modulated by the valence of the feedback: While in young adults, the FRN was equally pronounced for positive and negative feedback, older adults showed a significantly larger FRN amplitude to positive vs. negative feedback. Furthermore, when the feedback was inherently emotional in nature (i.e., faces with positive vs. negative emotional expressions), the P3b was larger for negative than for positive feedback in older adults.

### ERPs related to feedback processing and cognitive control in cognitive decline and SCD

Nitta et al. (2017) reported that, compared to healthy young and older adults, FRN amplitude in a gambling task was enhanced in patients with Alzheimer’s disease in general, and more strongly so for negative than positive feedback. This enhancement in FRN amplitude for negative feedback was correlated with depressive symptoms (Nitta et al., 2017). P3b amplitude, by contrast, was reduced in the patient group. A similar FRN enhancement has also been reported for patients with mild cognitive impairment (MCI; Abe et al., 2023). In light of these findings, previously reported reductions of FRN amplitude in older adults may not be a direct index of cognitive decline, but might index a motivational bias (cf. Ferdinand, 2019). Neurodegenerative conditions could thus be associated with a shift in this processing bias back from positive toward negative outcomes. If SCD represents and early stage of cognitive decline, one may thus expect a similar pattern in individuals with SCD.

Although some studies have examined correlates of SCD in EEG parameters (e.g., Babiloni et al., 2010; Mazzon et al., 2018), very few examined ERPs in cognitive control tasks, and the study designs and result patterns have been heterogeneous (Parker et al., 2022). Due to the scarcity of prior studies using the FRN in a similar design as the present study, we here additionally draw upon prior work that has examined morphologically and functionally related negativities like the MFN, NoGo-N2 and FRN, which presumably all reflect early cognitive control processes emerging in the anterior cingulate cortex (Folstein and Van Petten, 2008).

Three relevant studies (Garrido-Chaves et al., 2021) used questionnaire scores to divide participants into groups with and without subjective memory complaints (SMCs; a construct that strongly overlaps with SCD). Garrido-Chaves et al. (2021) reported that in an IOWA Gambling Task (IOT), a prolonged FRN latency was elicited in an older adult SMC group by negative feedback in the first task block. No amplitude differences were found in the FRN or P3b. Cespón et al. (2018) examined the medial frontal negativity (MFN), which is sensitive to response conflict, and the P300 in a Simon task. In the absence of overall group differences in P3b or MFN amplitude, MFN amplitude was differentially modulated by response conflict only in the SMC group. The authors interpreted this pattern as more resources being allocated to conflict monitoring in the SMC group. Third, Susana et al. (2021) examined the N2 and P3 in a Go-NoGo task. The SMC group showed slower responses and a reduction in both N2 and P3 amplitude, compared to the low-SMC group. The ERP effects overlapped with a preparatory negativity elicited by a warning tone, which was more pronounced in the low SMC group, potentially accounting for the N2 and P3 amplitude differences. The authors speculated that the high SMC group showed deficient allocation of resources to (preparatory) stimulus processing.

Smart et al. (2014) divided cognitively unobtrusive participants into a SCD and a no SCD group based on their answer on a question regarding worries about cognitive decline. In the absence of group differences in task performance in a Go/NoGo-task, the SCD group showed a reduced P3. Results for the N2 were not reported.

The results of these studies are hence heterogeneous and somewhat contradictory, suggesting enhanced or reduced negativities, or no group differences, in participants with SCD. Regarding the P3, some evidence points to a reduced amplitude in SCD, but not all studies have found this pattern. While any differences between studies could be in part due to the specific measures of cognitive complaints, clearly more evidence is needed to clarify the role of feedback processing in SCD on a neural level.

### The present study

To examine feedback processing in older adults with and without SCD, recruited from the community, we adopted the probabilistic feedback learning task from Ferdinand and Hilz (2020). We hypothesized a generally increased FRN amplitude, reflecting a hyperactivity of the monitoring system in SCD. Alternatively, a motivational processing bias, reflected in a relative enhancement of FRN amplitudes for negative, relative to positive feedback, may be observed in SCD. Regarding the P3b, as a marker of (very) early stages of cognitive decline, we hypothesized a reduced P3b amplitude, possibly with a more anterior scalp distribution, in older adults with SCD. Due to the fact that individuals with SCD do not show neuropsychological test performance below the age norms, we had no specific hypotheses regarding the learning rates.

## Methods

The present study was reviewed and approved by the ethics committee at Trier University. All participants provided informed consent.

### Sample and general procedure

Because of a larger interruption due to the COVID-19-pandemic, data collection occurred in two waves. Participants who were at least 64 years old and who self-reported to be generally in good health were recruited from the community. A history of neurological conditions affecting the central nervous system or current psychiatric conditions composed an exclusion criterion. All data sets were collected in a two-step procedure, the first step involving a packet of questionnaires at home and an on-site neuropsychological test session, and the second step involving a laboratory-based EEG-experiment including a probabilistic learning task. Participants completed this experimental session within 8 weeks of completing the questionnaires and neuropsychological testing. All measures and tasks reported in the present study were identical in both phases, but since there were minor changes in the general procedure, the procedure of each wave will be described briefly. It is also worth noting that there were no differences in the questionnaire or neuropsychological test scores, age or years of education between the subsamples collected in waves 1 and 2 (all *p*-values >0.073).

The first wave was conducted between June–August 2019. Fifty-three older adults (64 years or older), who were recruited from an existing data base, completed an unrelated behavioral memory experiment and completed the CERAD-plus (Consortium to Establish Registry for Alzheimer’s disease; Fillenbaum and Mohs, 2023). Furthermore, a questionnaire packet was filled out at home. Participants were next invited to a second session in which they completed the present study’s task. Based on their responses to the question “Do you notice a significant decline in your cognitive abilities, which exceeds the normal age-related decline?,” participants were divided into two groups. Participants were considered part of the SCD group only if they answered “Yes, and I am worried about this” (e.g., Ruiz-Rizzo et al., 2022). If one of the other two response options was chosen (“No” or “Yes, but I am not worried about this”), they were considered part of the No-SCD group. A total of *n* = 8 participants from the SCD group, and *n* = 22 participants from the No-SCD group returned for the present study. One additional No-SCD participant took part, but due to a technical error, no data were collected.

The second wave spanned January 2022–December 2023. Older adults who responded to calls for participation, distributed via newspapers and flyers, were mailed the same packet of questionnaires. All participants fulfilling the criterion for the SCD-group (defined in the same manner as for the first wave) were invited to the present study. For each SCD participant, we invited another participant who answered “No” to the critical question and was matched in gender and age (+/− 3 years). The CERAD-Plus was completed, together with an unrelated cognitive task, in a separate session preceding the present task. Eleven SCD- and 15 No-SCD participants took part in the present task. For two participants from the SCD-group, no EEG was recorded due to a technical error. One participant from the No-SCD group was excluded due to low EEG data quality. Hence, the second wave yielded *n* = 9 participants in the SCD-group and *n* = 14 participants in the No-SCD group.

The final sample hence consisted of *n* = 17 participants in the SCD-group and *n* = 36 participants in the No-SCD group. The two groups did not differ in age (Table 1, *p* = 0.46) or gender (chi-square test: *p* = 0.31). With a power of 1-*β* = 0.9 and *α* = 0.05, a sample of *N* = 53 has the sensitivity to detect a between-within interaction of a medium effect size of *f* = 0.23 in a 2×2 mixed factors ANOVA (Faul et al., 2007).

**Table 1:** Means (+/− SD) for demographic variables, neuropsychological test scores and questionnaires for the No-SCD and the SCD group.

	No-SCD (*n* = 36)	SCD (*n* = 17)
Demographic information		
Age	71.19 (4.73)	72.29 (5.38)
Sex	50.00% female	64.71% female
Years of education^1^	15.71 (3.51)	15.41 (3.57)
CERAD-Plus		
Semantic Fluency (Correct Words Total)	22.78 (5.43)	21.59 (5.27)
Boston Naming Test (Correct Words Total)	14.17 (0.96)	13.82 (1.29)
Mini Mental State Exam (Total)	28.64 (1.32)	28.29 (1.07)
Word List Learning (Sum over Trials 1–3)*	20.25 (3.68)	17.41 (3.34)
Figure Copying (Average across Trials 1–4)	10.47 (0.87)	10.59 (0.77)
Word List Recall: Correct Words	7.22 (2.38)	6.59 (1.82)
Word List Recognition: Correct Responses Total	19.94 (1.76)	19.53 (0.85)
Figure Recall (Average across Trials 1–5)	10.47 (2.39)	9.82 (2.50)
TMT A (completion time in sec)^†^	41.17 (15.90)	48.82 (15.90)
TMT B (completion time in sec)	111.83 (57.18)	113.71 (55.30)
Phonemic Fluency (Correct Words Total)	14.97 (5.08)	14.00 (6.04)
Questionnaires		
Neo-FFI-Neuroticism^†^	12.69 (3.43)	14.79 (4.22)
Neo-FFI-Extraversion	19.33 (3.20)	19.32 (3.04)
Neo-FFI-Openness	21.50 (3.82)	21.88 (4.36)
Neo-FFI-Agreeableness	23.47 (3.00)	21.91 (3.67)
Neo-FFI-Conscientiousness*	25.33 (3.05)	23.12 (2.59)
Depression (GDS)**	1.59 (1.70)	3.56 (2.15)
SCD**	9.17 (4.25)	15.59 (4.30)
Trait Anxiety (STAI)**	33.19 (6.94)	39.53 (8.40)
Response Accuracy		
First quarter	0.52 (0.07)	0.54 (0.07)
Second quarter	0.65 (0.11)	0.64 (0.09)
Third quarter	0.69 (0.13)	0.68 (0.11)
Fourth quarter	0.72 (0.15)	0.71 (0.13)
Event-related potential amplitudes (μV)		
Peak-to-peak FRN: positive feedback (FCz)	−13.24 (5.00)	−13.36 (6.88)
Peak-to-peak FRN: negative feedback (FCz)	−11.43 (4.37)	−12.84 (7.30)
P300: positive feedback (Pz)	4.04 (3.31)	3.79 (2.65)
P300: negative feedback (Pz)	6.02 (4.14)	4.96 (3.38)

*p < 0.05, **p < 0.01, ^†^*p* < 0.1, ^1^There were 2 missing values on years of education in the NoSCD-group.

Due to the fact that the SCD and the No-SCD group were not matched in size and hence, simple comparisons had a larger power in the No-SCD group, any simple contrasts for the No-SCD group, after the main analyses were completed, were repeated with a sub-sample that was the same size as the SCD group (*n* = 17) as a control analysis. For this purpose, for each participant in the SCD group we drew a specific participant of the same sex from the same data collection wave from the No-SCD group (which all had answered “No” to the critical question), such that each pair was as close in age as possible, with a maximal age difference of 3 years (except for 2 pairs in which a male was matched to a female due to a lack of other viable matches, which, however resulted in the same number of males and females in the two groups).

### Neuropsychological testing

All participants completed the CERAD-Plus (Consortium to Establish Registry for Alzheimer’s disease; Fillenbaum and Mohs, 2023) in individual sessions. The CERAD-Plus contains, in this order, (1) a *semantic fluency* task, in which as many animals should be named as possible in 1 min, (2) a 15-item version of the Boston Naming Test, (3) the Mini-Mental State Exam as a *screening for dementia* (MMSE), (4) a *word list learning* task including free recall after each of three presentations of a word list, (5) a *figure copying* task of figures, (6) a *delayed verbal recall* task for the words of the list learning task, (7) a *verbal recognition* task for the list learning task, (8) drawing of the 4 figures from the figure copying task as well as the figure from the MMSE from memory (i.e., *visuo-spatial recall*), (9) the trail making tests A and B as measures of *processing speed and executive function*, (10) a *phonemic fluency* task in which as many words starting with the letter “S” have to be produced in 1 min. The total duration of all tests contained in the CERAD-Plus was about 30 min.

### Questionnaires

Paper-based versions of all questionnaires were mailed to the participants and filled out by paper-and-pencil at home. The administered questionnaires included the following: The Neo-FFI-30 (German version; Körner et al., 2008) contains 30 items that capture neuroticism, openness, agreeableness, extraversion and conscientiousness with 6 items each; for each scale, we calculated the sum score across all items, each ranging from 1 to 5. The geriatric depression scale (GDS; Yesavage and Sheikh, 1986) contains 15 items to screen for depressive symptoms in older adults. The state-and-trait-anxiety-inventory (STAI; Spielberger et al., 1971) contains 20 items that can be administered to capture state or trait anxiety. In the present study, only the trait anxiety scale was used. The scales of the Neo-FFI (Körner et al., 2015), the GDS (Almeida and Almeida, 1999) and the STAI (Metzger, 1976) all show acceptable reliabilities and are well-validated instruments to measure the respective constructs. Further, we administered the SCD Questionnaire (Gifford et al., 2015), which contains 21 items that capture the subjective perception of everyday cognitive lapses, such as remembering phone numbers, translated to German. A sum score is calculated from all items, with higher scores reflecting a higher amount of SCD symptoms. The 21 items of the SCD Questionnaire have been developed based on item response theory and there is preliminary evidence for its reliability and validity in distinguishing between MCI patients and healthy controls (Gifford et al., 2015). A Chinese version has also been validated with MCI patients versus healthy controls (Hao et al., 2022). Finally, we added the question “Do you notice a significant decline in your cognitive abilities, which exceeds the normal age-related decline?” with three response options to the questionnaire package, which was used to determine each participants’ experimental group in a single step (SCD or No-SCD; see sample-description above). Note that this question was not included in the calculation of the score of the SCD Questionnaire. Several additional questionnaires were administered, which were unrelated to the present research question and are hence not reported.

### EEG session and probabilistic learning task

The session began with the preparation of the EEG recording, which took up to 45 min. Next, participants completed a probabilistic learning task, which was adopted from Ferdinand and Hilz (2020) and included only the “emotional” feedback category from the prior study. The total duration of the task was about 45 min. According to the cover story, the task was to sort objects (such as tools or animals) presented as color drawings (Snodgrass and Vanderwart, 1980) on the screen into a white or a black moving van, by pressing one of two buttons. The correct assignment of the objects to the vans had to be learned by utilizing probabilistic performance feedback, which was provided by the image of the face of a hypothetical supervisor. A happy facial expression signaled a correct response (positive feedback) and a disgusted facial expression signaled an incorrect response (negative feedback). The faces images showed one of four individuals, two of which were male and two of which were female. According to the cover story, the supervisors were likely to provide accurate feedback, but due to being busy with tasks regarding the move, in rare occasions they would make mistakes and give inaccurate feedback. Invalid feedback thus occurred in 10% of the trials and these trials were not analyzed.

The task began with a short practice phase, followed by four experimental blocks, in between which participants could take a self-paced break. In each experimental block, the assignment of eight objects to the two moving vans had to be learned. The objects were presented in a random order a total of 20 times each. A trial involved the presentation of the object image, to which participants had to press one of two buttons, representing the two moving vans. The presentation time of the objects was response-dependent, with a maximum of 1,500 ms. The object was next surrounded by a frame in the color of the chosen van for 500 ms. After a blank screen for 500 ms, a photo of a face was shown, which served as feedback regarding the accuracy of the response, and which was valid in 90%, but invalid in 10% of the trials. An adaptive response deadline was implemented: Participants began with a response deadline of 1,000 ms. If their response time exceeded this limit, the words “too slow” were shown after the stimulus. After each set of 19 trials, the response deadline was adjusted depending on the number of time outs within that set of trials. For further detail, see Ferdinand and Hilz (2020).

### EEG recording and analysis

The EEG recording was captured from 32 Ag/AgCl scalp electrodes embedded in an elastic cap according to the 10–20 electrode system, using a NeurOne Tesla amplifier (Bittium, Inc.). Additionally, an EKG was recorded and all signals were grounded to electrode position AFz. The EEG was amplified at 0.16–7,000 Hz, on-line filtered with a hardware lowpass filter of 125 Hz, and digitized at a rate of 500 Hz.

The signal was on-line referenced to electrode FCz and off-line re-referenced to the average of electrodes TP9 and TP10 (corresponding to an average mastoid reference). As a part of the re-referencing, the signal for electrode FCz was reconstructed. Off-line analysis was conducted using BrainVision Analyzer 2.2 (Brain Products). The EEG was filtered with a zero phase shift butterworth low-pass filter of 4th order, with a 20 Hz cutoff. Next, the data were segmented from −200 to 800 ms relative to the onset of the feedback display. Eye blinks and saccade artifacts were corrected using the semi-automatic ICA procedure, with the infomax algorithm, implemented in the BrainVision Analyzer. Segments in which the amplitude range exceeded 100 μV or in which a voltage step of 30 μV/ms occurred were marked as artifactual and were excluded. Finally, subject ERP averages were calculated separately for positive and negative feedback. Notably, trials with invalid feedback were not included in subject averaging.

Trial numbers in the final sample ranged from 20 to 290 (*M* = 146; No-SCD group) or 60–240 (*M* = 150; SCD group) for positive feedback and from 72 to 458 (*M* = 334; No-SCD group) or 252–427 (*M* = 332; SCD group) for negative feedback. There were no group differences in trial numbers (both *p*-values >0.82).

### Statistical analysis

Group differences in the questionnaire scores and the sub-tests of the CERAD-Plus were analyzed exploratorily with independent samples *t*-tests. The analyses of the experimental task were analogous to Ferdinand and Hilz (2020). Learning rates were quantified by calculating the average percentage of correct responses for each of four successive experimental blocks. A 2 (group: SCD vs. No-SCD) by 4 (experimental quarter) mixed factors ANOVA served to analyze learning rates.

The FRN was defined as the peak-to-peak amplitude between the positive peak between 170 and 230 ms and the negative peak between 250 and 350 ms at electrode FCz. Minor adjustments to the time widows, as compared to Ferdinand and Hilz (2020), were made after inspection of the grand averages across groups, ensuring that they adequately accounted for inter-individual variability in the latency of ERP peaks. The FRN was statistically analyzed with a 2 (group: SCD vs. No-SCD) × 2 (feedback valence) mixed factors ANOVA. In additional, exploratory control analyses, neuropsychological and questionnaire variables that differed between the two groups were added as covariates into a series of ANCOVAs to examine whether the obtained result pattern remained robust when these variables were statistically accounted for.

The P3b was analyzed at 3 midline electrodes (Fz, Cz, and Pz), and was quantified as the mean amplitude between 400 and 600 ms after feedback onset. The P3b was statistically analyzed with a 2 (group: SCD vs. No-SCD) × 2 (feedback valence) × 3 (electrode) mixed factors ANOVA.

## Results

### Questionnaires and neuropsychological tests scores

Table 1 summarizes demographic information, questionnaire scores, neuropsychological test scores, learning rates, and ERP amplitudes from the probabilistic learning task. As mentioned in the methods section, the two groups did not differ in age (Table 1), *t*(51) = 0.76, *p* = 0.45, education, *t*(51) = 0.33, *p* = 0.75, nor gender (chi-square test: *p* = 0.31). The No-SCD group showed better word list learning than the SCD group, *t*(51) = 33.85, *p* = 0.01, *d* = 0.78, but no significant differences in delayed word recall or any other neuropsychological test scores (all *p*-values >0.29). Furthermore, the SCD group exhibited significantly higher scores in trait anxiety (STAI), *t*(51) = 2.90, *p* = 0.005, *d* = 0.8, depression (GDS), *t*(51) = 3.52, *p* < 0.001, *d* = 1.04, and the SCD questionnaire, *t*(51) = 5.32, *p* < 0.001, *d* = 1.57, as well as lower scores on conscientiousness, *t*(51) = 2.54, *p* < 0.014, *d* = 0.75, compared to the No-SCD group.

### Learning rates

**Figure 1:**
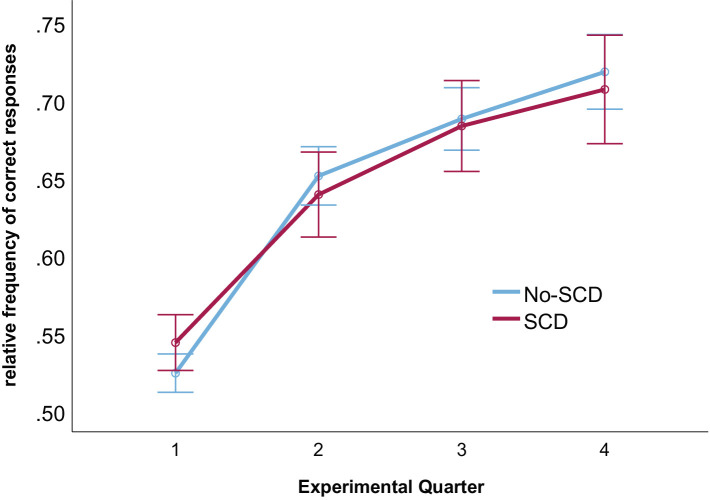
Rates of correct responses for each experimental quarter and for both groups. Error bars denote the standard error of the mean.

Learning rates are illustrated in Figure 1 (see also Table 1). A 2 (group: SCD vs. No-SCD) by 4 (experimental quarter) ANOVA on the rate of correct responses revealed a main effect for experimental quarter, *F*(3, 153) = 76.53, *p* < 0.001, *η*^2^_p_ = 0.60. Neither the interaction nor the main effect for group reached significance (both *p-*values >0.49). Comparing each successive pair of experimental quarters in lower-level ANOVAs, significant increases in the rate of accurate responses were observed between each pair (all *p*-values <0.003).

### FRN peak-to-peak amplitude

**Figure 2:**
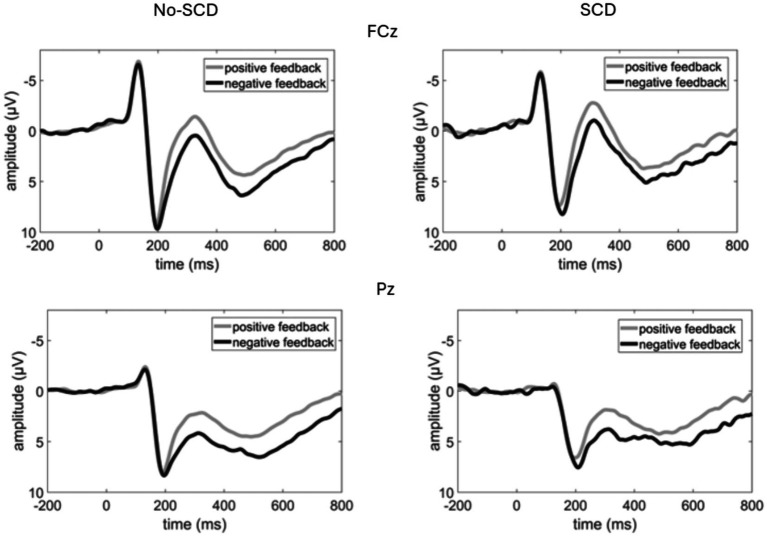
Event-related potential waveforms elicited by the feedback stimulus at a fronto-central (FCz) and a parietal electrode (Pz). Note that negative voltages are plotted upwards.

**Figure 3:**
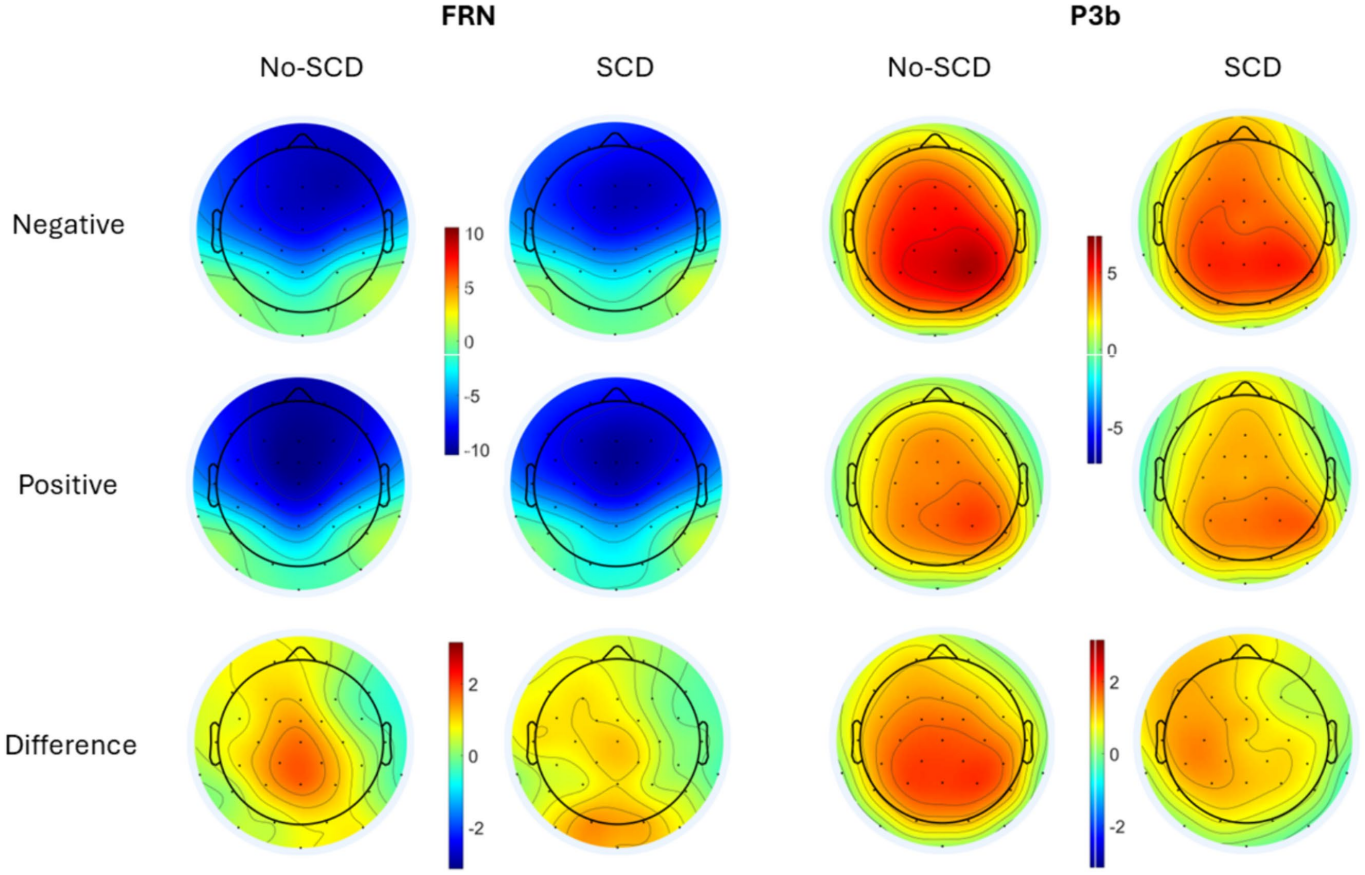
Scalp distributions of the peak-to-peak FRN **(Left)** and the P3b **(Right)** by group and feedback valence (positive vs. negative response). The difference is shown as negative–positive. Note that as the FRN is a peak-to-peak measure while the P3b is a mean amplitude measure, amplitudes from the two components cannot be directly compared to each other.

Grand average ERP waveforms are shown in Figure 2, and scalp distributions are shown in Figure 3. A 2 (group: SCD vs. No-SCD) by 2 (feedback valence: positive or negative) ANOVA on the peak-to-peak FRN revealed a significant main effect for feedback valence, *F*(1, 51) = 13.59, *p* < 0.001, *η*^2^_p_ = 0.21, indicating that positive feedback elicited a larger peak-to-peak FRN than negative feedback. There was also a significant group × feedback valence interaction, *F*(1, 51) = 4.13, *p* = 0.047, *η*^2^_p_ = 0.075, but no main effect for group (*p* = 0.64).

**Figure 4:**
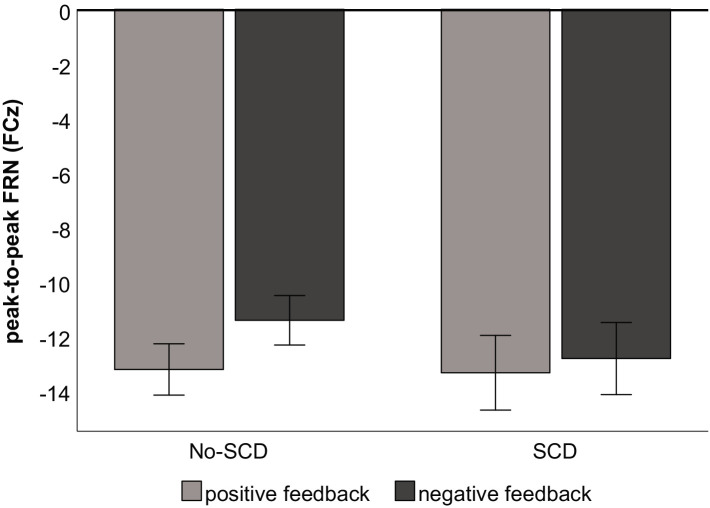
Peak-to-peak amplitude of the FRN by feedback type and group. Error bars denote the standard error of the mean.

In the SCD group there was not a significant difference between the FRN amplitudes elicited by positive vs. negative feedback, *t*(16) = 0.88, *p* = 0.39 (Figure 4). By contrast, for the No-SCD group, positive feedback elicited a significantly larger FRN than negative feedback, *t*(35) = 5.45, *p* < 0.001, *d* = 0.91 (Figure 4 and Table 1).

In the sub-sample of 17 No-SCD participants that were matched to the SCD group, the difference in FRN amplitude between positive and negative feedback was still highly significant, *t*(16) = 3.80, *p* = 0.002, *d* = 0.92. However, repeating the 2 (group) × 2 (feedback valence) ANOVA in the smaller sub-sample, while the main effect for valence was still significant, *F*(1, 32) = 7.26, *p* = 0.011, *η*^2^_p_ = 0.19, the interaction was not significant, *F*(1, 32) = 1.40, *p* = 0.25, *η*^2^_p_ = 0.042.

### P3b

The analysis of the P3b-time window yielded a main effect of electrode, *F*(1.62, 82.83) = 8.71, *p* < 0.001, *η*^2^_p_ = 0.146, indicating a posterior distribution, as well as a main effect for feedback valence, *F*(1, 51) = 23.56, *p* < 0.001, *η*^2^_p_ = 0.314, indicating that the P3b was larger for negative than for positive feedback (Figure 2 and Table 1). There was a tendency for an electrode × feedback interaction, which was, however, non-significant, *F*(1.41, 71.93) = 3.08, *p* = 0.069, *η*^2^_p_ = 0.057. The difference between positive and negative feedback tended to be larger at posterior than at frontal electrodes. There were no significant main or interaction effects involving the factor group. These results were equivalent when only the smaller subsample of No-SCD participants was considered.

### Control- and exploratory analyses

Several additional analyses were conducted *post hoc* to examine the robustness of, and mechanisms behind, the group x valence interaction on the FRN amplitude in the whole sample. First, in a series of ANCOVAs, we included each questionnaire and neuropsychological test score that differed between the groups (see section “Questionnaires and Neuropsychological Test Scores”) individually as covariates to examine whether the group × valence interaction remained when the influence of these variables was accounted for. When separately controlling for Anxiety (STAI), Neuroticism (NEO-FFI), and Processing Speed (CERAD-TMT A), the group × valence interaction effect on the peak-to-peak FRN in the ANCOVAs remained significant at *p* < 0.05. For Depression (GDS) and Conscientiousness (NEO-FFI), the interaction was numerically still pronounced but missed the significance level (both *p*-values <0.07).

When CERAD Word List Learning was entered as a covariate into an ANCOVA, the valence × group interaction was non-significant, *F*(1, 50) = 1.73, *p* = 0.20. We hence further examined a potential role of CERAD Word List Learning in the association of the group (SCD/No-SCD) with the valence effect on the FRN. To do so, we calculated a difference score between FRN amplitudes for positive and negative valences and tested whether CERAD word list learning mediated the effect of group on this difference score, using Process for SPSS, Version 4.2 (model 4). In this model, the standardized regression coefficient of −0.39 for the effect of group on the FRN difference score was non-significant (*p* = 0.19). However, group significantly predicted CERAD Word List learning (−0.74, *p* = 0.01), and higher CERAD scores tended to be associated with an increased FRN difference score (standardized regression coefficient: 0.26, *p* = 0.07). We tested the significance of the indirect effect with 5,000 bootstrap samples, and the unstandardized indirect effect of −0.4187 (95% CI -0.0161, −1.0337) was statistically significant. By contrast, the direct effect of group on the FRN difference was non-significant in this model. Hence, this analysis suggests that statistically, CERAD word list learning fully mediated the effect of the group (SCD/No-SCD) on the FRN difference between positive and negative feedback. A figure illustrating this mediation is provided at: https://osf.io/cf32m/.

## Discussion

To summarize our main results, the present community sample of older adults with SCD symptoms, in the absence of differences in performance in a probabilistic learning task, showed altered processing of negative vs. positive feedback in electrophysiological markers of neural activity. Specifically, the amplitude of the FRN was larger for positive than for negative feedback in the No-SCD, but not in the SCD group. While P3b amplitude was descriptively reduced in the SCD group, this difference was not significant. As we will discuss in the remainder of this article, the present results speak for a processing bias toward negative (versus positive) performance feedback in SCD.

### Sample characteristics

We drew participants from the community and operationalized SCD by means of the answer to a single question, which captured (1) the perception of cognitive decline beyond a normal age-related development and (2) a worry about this decline, following the SCD-plus criteria (Jessen et al., 2014). As expected, the two groups differed in their scores on the (independently captured) SCD questionnaire. The SCD group also showed typical characteristics in the other questionnaire measures, including enhanced scores in depression and anxiety, as well as lower scores on conscientiousness (as well as a tendency for higher scores in neuroticism). Except for a difference in the CERAD word list learning score, no group differences were found in neuropsychological test scores. Furthermore, there were no significant differences in demographic variables. In these aspects, we found the typical pattern for individuals with versus without SCD (Jessen et al., 2014). We will return to a discussion about the operationalization and measurement of SCD below.

### Comparison to Ferdinand and Hilz (2020)

The task used in the present study was adopted from Ferdinand and Hilz (2020), using only the “emotional feedback” condition of that prior study, so it is important to briefly discuss our results in comparison to this prior study. The present result pattern, including the learning rates across experimental quarters, the larger FRN for positive compared to negative feedback in the No-SCD group, and the enhanced P3b for negative feedback across both groups of older adults, was analogous to the results from the older adults in Ferdinand and Hilz (2020). Hence, the present results largely replicate those of this prior study. They indicate a strong monitoring process for positive feedback (FRN) in older adults when the feedback is emotional in nature, while working memory updating (P3b) in this learning task is driven mainly by negative (emotional) feedback.

### Altered FRN to negative vs. positive feedback in SCD

The main finding of the present study was a relative enhancement of FRN amplitude to negative, relative to positive, feedback in the SCD group. That is, while in the No-SCD group, the FRN was significantly larger for positive than for negative feedback, FRN amplitude did not differ by feedback valence in the SCD group. Importantly, there were no overall group differences in FRN amplitude (independently of feedback valence), suggesting that the differential FRN pattern in the two groups is not due to a generalized group difference in performance monitoring processes (such as a hyperactivity of monitoring systems *per se*). Comparable overall FRN amplitudes also suggest that a monitoring deficit, perhaps caused by a subtle decline in neural processing in the SCD group, is unlikely to directly account for the result pattern. The latter point is also supported by the fact that the FRN pattern for the SCD group (no difference of FRN amplitude by feedback valence) was actually more similar to that of the young adults in Ferdinand and Hilz (2020) than that of the No-SCD group. Finally, since no differences were found in learning rates within the probabilistic learning task in the present study, group differences in the extent to which negative vs. positive feedback was unexpected can also be tentatively ruled out (with the caveat that we did not check whether the participants’ subjective expectancies directly matched the objective frequencies).

Hence, the most likely explanation for the differential FRN pattern appears to be a processing bias of the SCD group toward negative performance feedback. Given the fact that groups with a tendency to preferentially process negative information like patients with depression and anxiety have been reported to show enhanced FRN amplitudes to negative feedback (Santesso et al., 2012; Keren et al., 2018; Tobias and Ito, 2021; but see Gao et al., 2024 for a different result), this is in line with the fact that the SCD group showed stronger symptoms of depression and anxiety than the No-SCD group (Table 1). Some indirect evidence for a role of depressive symptoms in the present result pattern comes from the fact that the group × valence interaction missed the significance level when statistically controlling for depressive symptoms. Another hint comes from the fact that, while the simple contrasts revealed the equivalent patterns in the smaller, matched sub-sample (i.e., the difference in FRN amplitude between positive and negative feedback remained significant in the smaller sub-sample of 17 No-SCD individuals), the group × valence interaction became non-significant when considering only the reduced sample. Since the reduced sample did not include any participants that reported noticing a decline, but not considering it worrisome, it is possible that the shift in FRN sensitivity to negative vs. positive feedback is specifically related to the worry-aspect of SCD, thus particularly distinguishing participants that worry, from those that do not, about cognitive decline. However, the non-significant interaction in the reduced sample could also be due to a reduced power. A larger, more homogeneous, sample would be necessary to shed further light on the role of affective factors in the present result patterns.

Notably, an exploratory analysis revealed that CERAD word list learning, which was the only neuropsychological test variable that differed between the groups, mediated the effect of group on the FRN difference due to valence. Statistically speaking, the groups hence differed in the sensitivity of the FRN to feedback valence via their word list learning ability. It is important to keep in mind that this analysis was not planned for *a priori* and these results should be regarded with caution. A replication of this finding is necessary before strong conclusions can be drawn. Furthermore, these results are purely correlational; inferences about a causal relationship cannot be drawn. Assuming that word list learning may be taken as an indicator of neuro-cognitive function (such that perhaps reduced word-list-learning scores could be a marker of subtle decline), this pattern may nevertheless somewhat speak for a role of measurable cognitive difficulties in the observed shift in valence sensitivity of the FRN. Further research is necessary to test this.

To sum up, one mechanism related to SCD may be a relative enhancement of FRN amplitude to negative, relative to positive, performance feedback, and this pattern may be related to depressive symptoms and worries. Although the cross-sectional design of the present study does not permit causal conclusions, one possibility is that a stronger relative tendency to process negative feedback is a precursor of the development of a worry about cognitive decline. Alternatively, worries of one’s own cognition declining may lead to a stronger sensitivity to the processing of negative versus positive feedback, in the present design reflected in an equally pronounced FRN to positive and negative feedback.

### Interpretation of the P3b results

Both groups showed a larger P3b for negative feedback, thus replicating Ferdinand and Hilz (2020). It thus appears that the processing bias between the SCD and the No-SCD group, depending on feedback valence, did not extend to later, higher-level feedback processing stages. Updating learned representations was thus engaged to a larger extent by feedback about an incorrect, compared to a correct response (see also Frank et al., 2005; Ferdinand, 2019).

Although there was a tendency for a reduced P3b amplitude in the SCD group, which aligns with our original hypothesis, this difference was not statistically significant. Furthermore, there was no evidence for an extended “frontal shift” for the SCD, compared to the No-SCD group. Some (Smart et al., 2014; Susana et al., 2021), but not all (Cespón et al., 2018; Garrido-Chaves et al., 2021) previous studies have shown a decline in P3b amplitude in individuals with subjective cognitive complaints. P3b amplitude has been suggested as a marker of reduced neural computational power in aging (van Dinteren et al., 2014) and different clinical conditions including dementias (Hedges et al., 2016), Parkinson’s disease (Xu et al., 2022), and schizophrenia (Jeon and Polich, 2003). Since individuals with SCD compose a heterogeneous population (e.g., Jessen et al., 2014), and given the possibility that P3b amplitude may be sensitive to actual (rather than perceived) neuro-cognitive dysfunction, a heterogeneity within the sample, including some individuals with an increased risk for dementia and some without, could have resulted in a reduced power to uncover group differences in P3b amplitude.

One speculation is that the FRN, elicited by negative vs. positive feedback, and the P3b show a stronger relative association of subgroups with pronounced worries (FRN) and those with subtle cognitive symptoms (P3b), respectively, in SCD. Thus, we found little evidence for reliable cognitive symptoms, in the sense of reduced scores in neuropsychological tests, in SCD, but the SCD group showed clear differences, compared to the No-SCD group, in mood symptoms like depression and anxiety. Notably, Perrotin et al. (2017) reported that SCD patients who had already sought out a medical exam for cognitive decline, but not a community sample of participants with SCD, showed a significant gray matter volume decline in several brain regions including the hippocampus (despite no differences in neuropsychological testing). Given the fact that we recruited participants from the community rather than from memory clinics, the present sample may hence show a comparably higher tendency toward the mood rather than neuro-cognitive symptoms of SCD. This may, in turn, account for the specific FRN pattern, but no reduction in P3b amplitude. Along this line of thought, it would be interesting for future studies to systematically examine whether those individuals who do show a reduced P3b amplitude in SCD are also those who will progress to eventually show cognitive decline in the future. A longitudinal design would be necessary to examine this.

### Limitations

Several limitations are worth noting regarding the operationalization of SCD in the present study. First, we assigned participants to the SCD-group based on two characteristics: A subjective perception of a cognitive decline beyond what is perceived as normal, and a worry about this decline. Our operationalization of the SCD group thus focused on a combination of these two factors. The No-SCD group was somewhat heterogeneous in that it included participants who noticed no cognitive decline at all and those that did notice a decline but were not worried about this. Although a control analysis on a subset of 17 No-SCD participants included only participants that did report not noticing any cognitive decline, this smaller sample leads to a lowered statistical power. Future studies may either focus solely on participants who report no cognitive decline whatsoever, or collect a sufficient number of participants to examine differences between all three groups (no perceived decline, perceived decline but no worries, perceived decline with worries), to obtain more homogeneous groups.

Moreover, we ignored other potentially important characteristics of the SCD construct. For example, we did not capture or account for the age of onset of these SCD symptoms, as is recommended by the SCD initiative (Jessen et al., 2014). Furthermore, we had no measure to examine the “accuracy” of each individual’s subjective experience of cognitive decline. This could be achieved by implementing third-party assessments by close relatives, or by capturing independent measures of an individual’s metamemory accuracy (Chapman et al., 2022), in future studies.

Furthermore, since we used a cross-sectional design, no causal conclusions are possible between the differences in SCD symptoms and the difference of FRN amplitude to negative versus positive feedback. The present results can hence not arbitrate between the idea that a relative processing bias toward negative feedback is a precursor of SCD symptoms or the idea that individuals with SCD, perhaps related to a negative mood or worries, pay closer attention to negative feedback. To explore this, it will be important for future studies to implement longitudinal designs, which would also allow for an analysis of a sub-group of individuals who proceed to develop cognitive decline versus those who do not.

It is also unclear which role specifics of our probabilistic learning task played in the observed result pattern. There were thus a few participants who did not improve their performance across the task quarters. Specifically, five participants (2 SCD and 3 No-SCD) showed learning slopes at or below 0. Hence, both groups were evidently composed of some participants who improved their performance across the task, and who were hence successful in learning the correct assignments to the two moving vans, and others that did not. The small sample size did not permit a subgroup analysis of only those participants with slopes above zero, which could be an important route for further research. Furthermore, it would be important to determine which role the relatively high overall task difficulty (mean accuracy of 0.71 in the last quarter of the task; Figure 1) played in our result pattern. Although there were no group differences in task performance and hence task performance is not confounded with the groups, it is still unclear whether the results would generalize to a task that differs from the present one in factors such as task difficulty, and consequently the expectancy of positive vs. negative feedback. Finally, it would be interesting to examine to what extent our results generalize to different kinds of tasks. For example, Garrido-Chaves et al. (2021) reported no differences in the FRN amplitude pattern between older adults with and without memory complaints in the Iowa Gambling Task. Although other aspects of the study design and sample differed between that study and ours, differences in the task demands could account for the partially discrepant result patterns. Further research is needed to examine this question.

## Conclusion

In a probabilistic learning task, in which participants had to learn from emotional feedback, despite no differences in behavioral performance, we found differences between a community sample of individuals with SCD symptoms, compared to a group without such symptoms, in the relative amplitude of the FRN to negative vs. positive feedback. One early marker that differs between older adults with vs. without SCD could hence be the manner in which positive and negative performance feedback from the environment (and/or from internal feedback cycles) is processed.

It will be important for further research to replicate our results with larger samples and to extend them to longitudinal designs. If the relative FRN amplitude to positive vs. negative performance feedback is confirmed to be a robust SCD marker and potentially predictive of cognitive decline, a next step would be to examine its utility in improving early diagnosis of cognitive decline. Compared to other neuroimaging methods like fMRI, EEG is a relatively cost-effective technique that is relatively simple to apply. Hence, ERP measures are in principle suitable to be integrated into early diagnostic procedures of cognitive decline.

## Data availability statement

The datasets presented in this study can be found in online repositories. The names of the repository/repositories and accession number(s) can be found below: https://osf.io/cf32m/.

## Ethics statement

The studies involving humans were approved by Ethics committee of Trier University. The studies were conducted in accordance with the local legislation and institutional requirements. The participants provided their written informed consent to participate in this study.

## Author contributions

S-MK: Writing – review & editing, Visualization, Validation, Supervision, Resources, Project administration, Methodology, Investigation, Funding acquisition, Formal analysis, Data curation, Conceptualization. RE: Writing – review & editing, Methodology, Investigation, Data curation. LK: Writing – review & editing, Project administration, Investigation, Data curation. NF: Writing – review & editing, Writing – original draft, Software, Methodology, Conceptualization.

## References

[ref1] Abe S. Onoda K. Takamura M. Nitta E. Nagai A. Yamaguchi S. (2023). Altered feedback-related negativity in mild cognitive impairment. Brain Sci. 13:203. doi: 10.3390/brainsci13020203, PMID: 36831745 PMC9953936

[ref2] Almeida O. P. Almeida S. A. (1999). Short versions of the geriatric depression scale: a study of their validity for the diagnosis of a major depressive episode according to ICD-10 and DSM-IV. Int. J. Geriatr. Psychiatry 14, 858–865. doi: 10.1002/(SICI)1099-1166(199910)14:10<858::AID-GPS35>3.0.CO;2-8 10521885

[ref3] Babiloni C. Visser P. J. Frisoni G. De Deyn P. P. Bresciani L. Jelic V.. (2010). Cortical sources of resting EEG rhythms in mild cognitive impairment and subjective memory complaint. Neurobiol. Aging 31, 1787–1798. doi: 10.1016/j.neurobiolaging.2008.09.020, PMID: 19027196

[ref4] Buckley R. F. Laming G. Chen L. P. E. Crole A. Hester R. (2016). Assessing error awareness as a mediator of the relationship between subjective concerns and cognitive performance in older adults. PLoS One 11:e0166315. doi: 10.1371/journal.pone.0166315, PMID: 27832173 PMC5104449

[ref5] Cespón J. Galdo-Álvarez S. Díaz F. (2018). Event-related potentials reveal altered executive control activity in healthy elderly with subjective memory complaints. Front. Hum. Neurosci. 12:445. doi: 10.3389/fnhum.2018.00445 30487741 PMC6246637

[ref6] Chapman S. Joyce J. L. Barker M. S. Sunderaraman P. Rizer S. Huey E. D.. (2022). Subjective cognitive decline is more accurate when metamemory is better. Front. Aging Neurosci. 14:787552. doi: 10.3389/fnagi.2022.787552, PMID: 35370602 PMC8965471

[ref7] Cutler J. Apps M. A. J. Lockwood P. (2022). Reward processing and reinforcement learning: from adolescence to aging. [Epubh ahead of print]. doi: 10.31234/osf.io/pnuk8

[ref8] Dillen K. N. Jacobs H. I. Kukolja J. Richter N. von Reutern B. Onur Ö. A.. (2017). Functional disintegration of the default mode network in prodromal Alzheimer’s disease. J. Alzheimers Dis. 59, 169–187. doi: 10.3233/JAD-161120, PMID: 28598839

[ref9] Donchin E. (1981). Surprise!… surprise? Psychophysiology 18, 493–513. doi: 10.1111/j.1469-8986.1981.tb01815.x 7280146

[ref10] Dumas J. A. Kutz A. M. McDonald B. C. Naylor M. R. Pfaff A. C. Saykin A. J.. (2013). Increased working memory-related brain activity in middle-aged women with cognitive complaints. Neurobiol. Aging 34, 1145–1147. doi: 10.1016/j.neurobiolaging.2012.08.013, PMID: 23036586 PMC3540200

[ref11] Eppinger B. Kray J. Mock B. Mecklinger A. (2008). Better or worse than expected? Aging, learning, and the ERN. Neuropsychologia 46, 521–539. doi: 10.1016/j.neuropsychologia.2007.09.001 17936313

[ref12] Faul F. Erdfelder E. Lang A. G. Buchner A. (2007). G* power 3: a flexible statistical power analysis program for the social, behavioral, and biomedical sciences. Behav. Res. Methods 39, 175–191. doi: 10.3758/BF03193146, PMID: 17695343

[ref13] Ferdinand N. K. (2019). The influence of task complexity and information value on feedback processing in younger and older adults: no evidence for a positivity bias during feedback-induced learning in older adults. Brain Res. 1717, 74–85. doi: 10.1016/j.brainres.2019.04.011, PMID: 30991040

[ref14] Ferdinand N. K. Czernochowski D. (2018). Motivational influences on performance monitoring and cognitive control across the adult lifespan. Front. Psychol. 9:1018. doi: 10.3389/fpsyg.2018.01018, PMID: 29997541 PMC6028708

[ref15] Ferdinand N. K. Hilz M. (2020). Emotional feedback ameliorates older adults’ feedback-induced learning. PLoS One 15:e0231964. doi: 10.1371/journal.pone.0231964, PMID: 32352992 PMC7192411

[ref16] Ferdinand N. K. Kray J. (2013). Age-related changes in processing positive and negative feedback: is there a positivity effect for older adults? Biol. Psychol. 94, 235–241. doi: 10.1016/j.biopsycho.2013.07.006, PMID: 23886960

[ref17] Ferdinand N. K. Mecklinger A. Kray J. Gehring W. J. (2012). The processing of unexpected positive response outcomes in the mediofrontal cortex. J. Neurosci. 32, 12087–12092. doi: 10.1523/JNEUROSCI.1410-12.2012, PMID: 22933792 PMC6621524

[ref18] Fillenbaum G. G. Mohs R. (2023). CERAD (consortium to establish a registry for Alzheimer’s disease) neuropsychology assessment battery: 35 years and counting. J. Alzheimers Dis. 93, 1–27. doi: 10.3233/JAD-230026 36938738 PMC10175144

[ref19] Folstein J. R. Van Petten C. (2008). Influence of cognitive control and mismatch on the N2 component of the ERP: a review. Psychophysiology 45, 152–170. doi: 10.1111/j.1469-8986.2007.00602.x, PMID: 17850238 PMC2365910

[ref20] Frank M. J. Woroch B. S. Curran T. (2005). Error-related negativity predicts reinforcement learning and conflict biases. Neuron 47, 495–501. doi: 10.1016/j.neuron.2005.06.020, PMID: 16102533

[ref21] Gao Y. Panier L. Y. Gameroff M. J. Auerbach R. P. Posner J. Weissman M. M.. (2024). Feedback negativity and feedback-related P3 in individuals at risk for depression: comparing surface potentials and current source densities. Psychophysiology 61:e14444. doi: 10.1111/psyp.14444, PMID: 37740325

[ref22] Garrido-Chaves R. Perez V. Perez-Alarcón M. Crespo-Sanmiguel I. Paiva T. O. Hidalgo V.. (2021). Subjective memory complaints and decision making in young and older adults: an event-related potential study. Front. Aging Neurosci. 13:695275. doi: 10.3389/fnagi.2021.695275 34803649 PMC8595984

[ref23] Gifford K. A. Liu D. Romano R. R. Jones R. N. Jefferson A. L. (2015). Development of a subjective cognitive decline questionnaire using item response theory: a pilot study. Alzheimers Dement. 1, 429–439. doi: 10.1016/j.dadm.2015.09.004, PMID: 26878034 PMC4750048

[ref24] Hao L. Jia J. Xing Y. Han Y. (2022). The reliability and validity test of subjective cognitive decline questionnaire 21 with population in a Chinese community. Brain Behav. 12:e2709. doi: 10.1002/brb3.2709, PMID: 35866228 PMC9392547

[ref25] Hayes J. M. Tang L. Viviano R. P. van Rooden S. Ofen N. Damoiseaux J. S. (2017). Subjective memory complaints are associated with brain activation supporting successful memory encoding. Neurobiol. Aging 60, 71–80. doi: 10.1016/j.neurobiolaging.2017.08.015, PMID: 28923533 PMC6378370

[ref26] Hedges D. Janis R. Mickelson S. Keith C. Bennett D. Brown B. L. (2016). P300 amplitude in Alzheimer’s disease: a meta-analysis and meta-regression. Clin. EEG Neurosci. 47, 48–55. doi: 10.1177/1550059414550567 25253434

[ref27] Jeon Y. W. Polich J. (2003). Meta-analysis of P300 and schizophrenia: patients, paradigms, and practical implications. Psychophysiology 40, 684–701. doi: 10.1111/1469-8986.00070, PMID: 14696723

[ref28] Jessen F. Amariglio R. E. Van Boxtel M. Breteler M. Ceccaldi M. Chételat G.. (2014). A conceptual framework for research on subjective cognitive decline in preclinical Alzheimer's disease. Alzheimers Dement. 10, 844–852. doi: 10.1016/j.jalz.2014.01.001, PMID: 24798886 PMC4317324

[ref29] Kamp S. M. (2020). Preceding stimulus sequence effects on the oddball-P300 in young and healthy older adults. Psychophysiology 57:e13593. doi: 10.1111/psyp.13593, PMID: 32369200

[ref30] Keren H. O’Callaghan G. Vidal-Ribas P. Buzzell G. A. Brotman M. A. Leibenluft E.. (2018). Reward processing in depression: a conceptual and meta-analytic review across fMRI and EEG studies. Am. J. Psychiatry 175, 1111–1120. doi: 10.1176/appi.ajp.2018.17101124, PMID: 29921146 PMC6345602

[ref31] Klawohn J. Santopetro N. J. Meyer A. Hajcak G. (2020). Reduced P300 in depression: evidence from a flanker task and impact on ERN, CRN, and Pe. Psychophysiology 57:e13520. doi: 10.1111/psyp.13520, PMID: 31898810

[ref32] Körner A. Czajkowska Z. Albani C. Drapeau M. Geyer M. Braehler E. (2015). Efficient and valid assessment of personality traits: population norms of a brief version of the NEO five-factor inventory (NEO-FFI). Arch. Psychiatry Psych. 17, 21–32. doi: 10.12740/APP/36086

[ref33] Körner A. Geyer M. Roth M. Drapeau M. Schmutzer G. Albani C.. (2008). Persönlichkeitsdiagnostik mit dem neo-fünf-faktoren-inventar: Die 30-item-kurzversion (neo-ffi-30). PPmP 58, 238–245. doi: 10.1055/s-2007-986199, PMID: 17899495

[ref34] Mazzon G. De Dea F. Cattaruzza T. Manganotti P. Monti F. Accardo A. (2018). Memorization test and resting state EEG components in mild and subjective cognitive impairment. Curr. Alzheimer Res. 15, 809–819. doi: 10.2174/1567205015666180427114520, PMID: 29701152

[ref35] Meadows C. C. Gable P. A. Lohse K. R. Miller M. W. (2016). The effects of reward magnitude on reward processing: an averaged and single trial event-related potential study. Biol. Psychol. 118, 154–160. doi: 10.1016/j.biopsycho.2016.06.002, PMID: 27288743

[ref36] Mendonça M. D. Alves L. Bugalho P. (2016). From subjective cognitive complaints to dementia: who is at risk?: a systematic review. Am. J. Alzheimers Dis. Other Dement. 31, 105–114. doi: 10.1177/1533317515592331, PMID: 26142292 PMC10852868

[ref37] Metzger R. L. (1976). A reliability and validity study of the state-trait anxiety inventory. J. Clin. Psychol. 32, 276–278. doi: 10.1002/1097-4679(197604)32:2<276::AID-JCLP2270320215>3.0.CO;2-G

[ref38] Mizuno A. Ly M. Aizenstein H. J. (2018). A homeostatic model of subjective cognitive decline. Brain Sci. 8:228. doi: 10.3390/brainsci8120228, PMID: 30572628 PMC6316074

[ref39] Nitta E. Onoda K. Ishitobi F. Okazaki R. Mishima S. Nagai A.. (2017). Enhanced feedback-related negativity in Alzheimer’s disease. Front. Hum. Neurosci. 11:179. doi: 10.3389/fnhum.2017.00179, PMID: 28503138 PMC5408015

[ref40] Oliveira F. T. McDonald J. J. Goodman D. (2007). Performance monitoring in the anterior cingulate is not all error related: expectancy deviation and the representation of action-outcome associations. J. Cogn. Neurosci. 19, 1994–2004. doi: 10.1162/jocn.2007.19.12.1994, PMID: 17892382

[ref41] Parker A. F. Ohlhauser L. Scarapicchia V. Smart C. M. Szoeke C. Gawryluk J. R. (2022). A systematic review of neuroimaging studies comparing individuals with subjective cognitive decline to healthy controls. J. Alzheimers Dis. 86, 1545–1567. doi: 10.3233/JAD-215249, PMID: 35253749

[ref42] Perrotin A. La Joie R. de La Sayette V. Barré L. Mézenge F. Mutlu J.. (2017). Subjective cognitive decline in cognitively normal elders from the community or from a memory clinic: differential affective and imaging correlates. Alzheimers Dement. 13, 550–560. doi: 10.1016/j.jalz.2016.08.011, PMID: 27693187

[ref43] Pietschmann M. Endrass T. Czerwon B. Kathmann N. (2011). Aging, probabilistic learning and performance monitoring. Biol. Psychol. 86, 74–82. doi: 10.1016/j.biopsycho.2010.10.009, PMID: 21056080

[ref44] Ruiz-Rizzo A. L. Pruitt P. J. Finke K. Müller H. J. Damoiseaux J. S. (2022). Lower-resolution retrieval of scenes in older adults with subjective cognitive decline. Arch. Clin. Neuropsychol. 37, 408–422. doi: 10.1093/arclin/acab061, PMID: 34342647 PMC8865194

[ref45] Sambrook T. D. Goslin J. (2015). A neural reward prediction error revealed by a meta-analysis of ERPs using great grand averages. Psychol. Bull. 141, 213–235. doi: 10.1037/bul0000006, PMID: 25495239

[ref46] San Martín R. (2012). Event-related potential studies of outcome processing and feedback-guided learning. Front. Hum. Neurosci. 6:304. doi: 10.3389/fnhum.2012.00304 23162451 PMC3491353

[ref47] Santesso D. L. Bogdan R. Birk J. L. Goetz E. L. Holmes A. J. Pizzagalli D. A. (2012). Neural responses to negative feedback are related to negative emotionality in healthy adults. Soc. Cogn. Affect. Neurosci. 7, 794–803. doi: 10.1093/scan/nsr054, PMID: 21917847 PMC3475354

[ref48] Severo M. C. Paul K. Walentowska W. Moors A. Pourtois G. (2020). Neurophysiological evidence for evaluative feedback processing depending on goal relevance. NeuroImage 215:116857. doi: 10.1016/j.neuroimage.2020.116857, PMID: 32304885

[ref49] Smart C. M. Karr J. E. Areshenkoff C. N. Rabin L. A. Hudon C. Gates N.. (2017). Non-pharmacologic interventions for older adults with subjective cognitive decline: systematic review, meta-analysis, and preliminary recommendations. Neuropsychol. Rev. 27, 245–257. doi: 10.1007/s11065-017-9342-8 28271346

[ref50] Smart C. M. Segalowitz S. J. Mulligan B. P. MacDonald S. W. (2014). Attention capacity and self-report of subjective cognitive decline: a P3 ERP study. Biol. Psychol. 103, 144–151. doi: 10.1016/j.biopsycho.2014.08.016, PMID: 25204705

[ref51] Snodgrass J. G. Vanderwart M. (1980). A standardized set of 260 pictures: norms for name agreement, image agreement, familiarity, and visual complexity. J. Exp. Psychol. Hum. Learn. Mem. 6, 174–215. PMID: 7373248 10.1037//0278-7393.6.2.174

[ref52] Spielberger C. D. Gonzalez-Reigosa F. Martinez-Urrutia A. Natalicio L. F. Natalicio D. S. (1971). The state-trait anxiety inventory. Rev. Interam. Psicol. 5, 174–215.

[ref1001] Stewardson H. J. Sambrook T. D. (2020). Evidence for parietal reward prediction errors using great grand average meta-analysis. Int J Psychophysiol. 152, 81–86., PMID: 32272127 10.1016/j.ijpsycho.2020.03.002

[ref53] Susana C. F. Mónica L. Fernando D. (2021). Event-related brain potential indexes provide evidence for some decline in healthy people with subjective memory complaints during target evaluation and response inhibition processing. Neurobiol. Learn. Mem. 182:107450. doi: 10.1016/j.nlm.2021.107450, PMID: 33933631

[ref54] Tobias M. R. Ito T. A. (2021). Anxiety increases sensitivity to errors and negative feedback over time. Biol. Psychol. 162:108092. doi: 10.1016/j.biopsycho.2021.108092, PMID: 33865907 PMC8187315

[ref55] van Dinteren R. Arns M. Jongsma M. L. Kessels R. P. (2014). P300 development across the lifespan: a systematic review and meta-analysis. PLoS One 9:e87347. doi: 10.1371/journal.pone.0087347, PMID: 24551055 PMC3923761

[ref56] Verfaillie S. C. Binette A. P. Vachon-Presseau E. Tabrizi S. Savard M. Bellec P.. (2018). Subjective cognitive decline is associated with altered default mode network connectivity in individuals with a family history of Alzheimer’s disease. Biol. Psychiatry Cogn. Neurosci. Neuroimaging 3, 463–472. doi: 10.1016/j.bpsc.2017.11.012, PMID: 29735156

[ref57] Viviano R. P. Damoiseaux J. S. (2020). Functional neuroimaging in subjective cognitive decline: current status and a research path forward. Alzheimers Res. Ther. 12, 1–18. doi: 10.1186/s13195-020-00591-9 PMC7063727 32151277

[ref58] Walsh M. M. Anderson J. R. (2012). Learning from experience: event-related potential correlates of reward processing, neural adaptation, and behavioral choice. Neurosci. Biobehav. Rev. 36, 1870–1884. doi: 10.1016/j.neubiorev.2012.05.008, PMID: 22683741 PMC3432149

[ref59] West R. Huet A. (2020). The effect of aging on the ERP correlates of feedback processing in the probabilistic selection task. Brain Sci. 10:40. doi: 10.3390/brainsci10010040, PMID: 31936648 PMC7016596

[ref60] Xu H. Gu L. Zhang S. Wu Y. Wei X. Wang C.. (2022). N200 and P300 component changes in Parkinson’s disease: a meta-analysis. Neurol. Sci. 43, 6719–6730. doi: 10.1007/s10072-022-06348-6, PMID: 35982362

[ref61] Yesavage J. A. Sheikh J. I. (1986). 9/geriatric depression scale (GDS) recent evidence and development of a shorter version. Clin. Gerontol. 5, 165–173. doi: 10.1300/J018v05n01_09

